# Phylogenetic Diversity, Antibiotic Resistance, and Virulence of *Escherichia coli* Strains from Urinary Tract Infections in Algeria

**DOI:** 10.3390/antibiotics13080773

**Published:** 2024-08-15

**Authors:** Anfal Kara, Chiara Massaro, Giovanni M. Giammanco, Rosa Alduina, Naouel Boussoualim

**Affiliations:** 1 Laboratory of Applied Biochemistry, Faculty of Nature and Life Sciences, University Ferhat Abbas of Setif 1, Setif 19000, Algeria; karaanfel98@gmail.com (A.K.); naouel_24@yahoo.fr (N.B.); 2 Department of Biological, Chemical and Pharmaceutical Sciences and Technologies (STEBICEF), University of Palermo, Viale delle Scienze, Bldg. 16, 90128 Palermo, Italy; chiara.massaro01@unipa.it; 3 Department of Health Promotion, Mother and Child Care, Internal Medicine and Medical Specialties “G. D’Alessandro”, University of Palermo, 90127 Palermo, Italy; giovanni.giammanco@unipa.it; 4 NBFC, National Biodiversity Future Center, Piazza Marina 61, 90133 Palermo, Italy

**Keywords:** *Escherichia coli*, antibiotic resistance, biofilm, hemolysin, urinary tract infections, phylogenetic groups, imipenem

## Abstract

Urinary tract infections (UTIs) caused by *Escherichia coli* represent a significant public health concern due to the high virulence and antimicrobial resistance exhibited by these pathogens. This study aimed to analyze the phylogenetic diversity and antibiotic resistance profiles of Uropathogenic *E. coli* (UPEC) strains isolated from UTI patients in Algeria, focusing on virulence factors such as extended β-lactamase (ESBL) production, biofilm formation, and hemolytic activity. Phylogenetic grouping of 86 clinical imipenem resistant *E. coli* isolates showed the prevalence of group B2 (48.9%), followed by groups E (22.1%), unknown (12.8%), A (8.1%), and B1 (4.7%), and Clade I, D, Clade I, or Clade II (1.2%). The highest resistance rates were observed towards amoxicillin (86.04%), ticarcillin (82.55%), piperacillin (73.25%), nitrofurantoin (84.88%), and trimethoprim-sulfamethoxazole (51.16%). Notably, 69.8% of UPEC strains were multidrug-resistant (MDR) and 23.2% were extensively drug-resistant (XDR). Additionally, 48.9%, 42%, and 71% of strains demonstrated ESBL production, hemolytic activity, and weak biofilm production, respectively. Continuous monitoring and characterization of UPEC strains are essential to track the spread of the most resistant and virulent phylogenetic groups over time, facilitating rapid therapeutic decisions to treat infections and prevent the emergence of new resistant organisms, helping choose the most effective antibiotics and reducing treatment failure.

## 1. Introduction

*Escherichia coli*, a Gram-negative bacillus of the Enterobacterales family, is commonly found in the gastrointestinal tract of humans and various animals [1,2,3]. This organism is among the most significant and common species of the *Escherichia* genus in veterinary and medical fields [4] and is responsible for approximately 80–90% of infections [5]. Apart from intestinal diseases that *E. coli* can cause, the species possesses a high potential to cause extra-intestinal diseases, including urinary tract infections (UTIs), various intra-abdominal, pulmonary, skin, and soft tissue infections, neonatal meningitis, and bacteremia [2,6,7,8].

Phenotypic and genotypic characteristics are used to identify *E. coli* pathogenic strains or pathovars [1]. The definition of these pathotypes can be based on various criteria, such as the target organ, the infected host, the association with the targeted organs, the pathology caused by the strains, and the presence of a specific gene or genes alone or in combination [2]. In addition to the pathotype and pathovar, the classification of *E. coli* strains has been based on phylogenetic relationships [1,3].

Clermont et al. optimized a quadruplex polymerase chain reaction (PCR) to classify extracellular *E. coli* strains into eight phylogenetic groups: B2, B1, A, D, F, E, C, and clade I [1,7,9]. Commensal strains are primarily related to groups A and B1 and can be responsible for intestinal infections [10], while pathogenic strains primarily belong to groups B2 and D [4,5,9,11]. Moreover, the detection of phylogenetic groups plays an important role not only in understanding the populations of *E. coli* but also in clarifying the relationship between strains and diseases [12]. *E. coli* isolates can be distinguished in terms of characteristics such as patterns of antibiotic resistance, virulence genes, the use of sugars, and environmental characteristics [5,13]. Furthermore, several studies showed that regional variations in *E. coli* populations may exist due to differences in environmental factors, human population dynamics, and ecological conditions [14,15,16].

The global spread of multidrug-resistant (MDR) and extensively drug-resistant (XDR) *E. coli* strains has become a public health threat and a major concern worldwide [3]. Limited treatment options may complicate UTIs and their treatment and increase morbidity and mortality [3,17].

In this study, we analyzed 86 imipenem-resistant uropathogenic *E. coli* (UPEC) isolated from inpatients and outpatients with UPEC-associated urinary tract infections in northern Algerian populations. The isolates were analyzed for their phylogenetic groups, as well as antibiotic resistance patterns associated with virulence profiles such as extended β-lactamase production, biofilm formation, and hemolytic activity to characterize the resistance phenotype and to investigate the virulence factors associated with this type of resistance.

## 2. Results

### 2.1. Demographic Characteristics

A total of 86 urinary imipenem-resistant *E. coli* isolates were collected from several geographical locations around Algeria’s north-eastern province of Sétif: 20.9% (18 isolates) from the capital of the province (Sétif), 50% (43 isolates) from southwest of Sétif (Aïn Oulmène city), 14% (12 isolates) from extreme north (Bouandas city), 7% (six isolates) from the north (Tizi N’Bechar city), 8.1% (seven isolates) from the east (El-Eulma city). Of the total samples, 59.3% were collected from women and 40.7% from men (ratio of female/male: 51/35 = 1.45; *p* = 0.084), the ages of the patients ranged between 2 years and 92 years with a mean age of 36.19 years. Females were older (mean age: 39.4) than males mean age: 31.5), with statistical significance (*p* value < 0.001, *t* test). Most UPEC isolates were found in adults (*p* < 0.001), indicating they are more susceptible than other groups to UPEC UTIs. Table 1 shows the distribution of demographic characteristics of inpatients (hospitalized), or outpatients (day hospital visits) based on their gender and age.

Regarding the age group distribution, female patients (44.1%) in the 15–64 age class were prevalent compared to males (12.7%), especially in outpatients. However, in the children age group (<15 years), there were more males (19.7%) than females (6.9%) in both inpatient and outpatient settings. The elderly included in the study were only outpatients and there was no significant gender difference. The statistical analyses (Fisher’s exact test, *p* < 0.0001) indicated a strong and significant correlation between the age group and gender distribution of the participants in this study.

### 2.2. Phylogenetic Grouping

**Figure 1:**
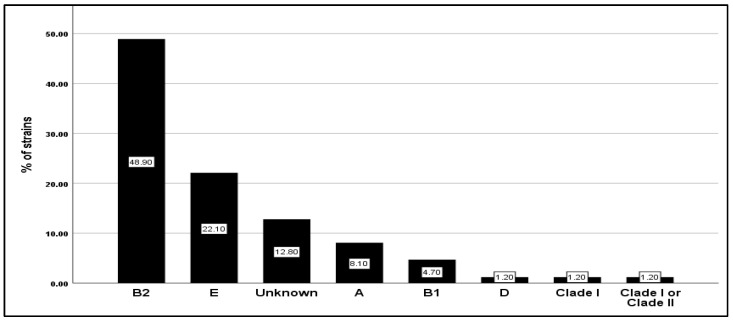
Frequency distribution of the 86 *E. coli* clinical isolates in seven phylogroups (A, B1, B2, D, E, Unknown, Clade I, and Clade I or Clade II).

Based on the quadruplex PCR assay, phylogenetic analysis of *E. coli* isolates showed that they mainly belonged to phylogroup B2 (48.9%) and E (22.1%), followed by A (8.1%), B1 (4.7%), and D, Clade I, Clade I or Clade II (1.2% for each one). Typing profiles that did not cluster with any of the known groups were frequently found (unknown groups; 12.8%), but no strain belonging to the phylogroup F was found (Figure 1). However, the statistical analyses did not reveal any significant correlation between the gender, the age of the patient, and the phylogenetic groups (*p* = 0.578 and 0.171, respectively).

### 2.3. Antimicrobial Susceptibility

**Figure 2:**
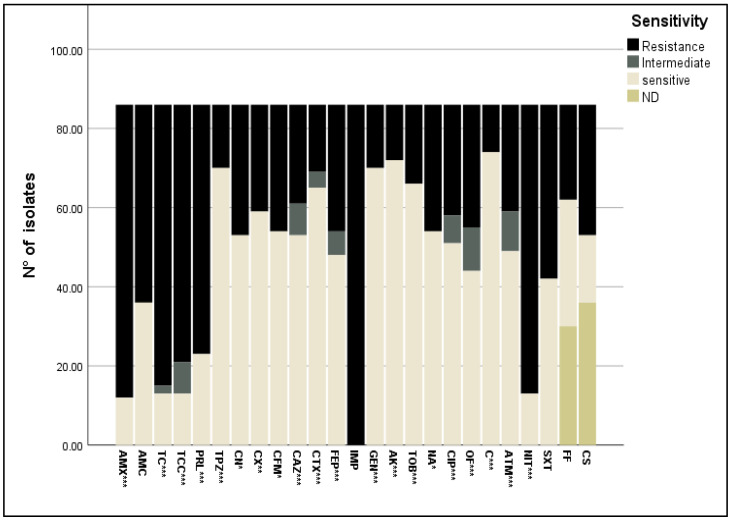
Antibiotic resistance profile of *E. coli* isolates towards 25 antibiotics belonging to nine classes. *: *p* < 0.05; **: *p* < 0.01; ***: *p* < 0.0001. AMX: amoxicillin, AMC: amoxicillin + clavulanic acid, TC: ticarcillin, TCC; ticarcillin + clavulanic acid, PRL: piperacillin, TPZ: piperacillin + tazobactam, CN: cephalexin, CX: cefoxitin, CFM: cefixime, CAZ: ceftazidime, CTX: cefotaxime, FEP: cefipime, IMP: imipenem, GEN: gentamycin, AK: amikacin, TOB: tobramycin, NA: nalidixic acid, CIP: ciprofloxacin, OF: ofloxacin, C: chloramphenicol, ATM: aztreonam, NIT: nitrofurantoin, SXT: trimethoprim-sulfamethoxazole, FF: fosfomycin, CS: colistin, ND: not determined.

The 86 *E. coli* isolates showed different resistance profiles towards the 25 antibiotics tested. Apart from the resistance to imipenem (IMP), the highest resistance percentage was significantly (*p* < 0.0001) observed against β-lactam antibiotics: amoxicillin (AMX) (86.04%) and ticarcillin (TC) (82.55%), followed by piperacillin (PRL) (73.25%). The association of β-lactamase inhibitors with β-lactam antibiotics was also investigated; the association of the β-lactamase inhibitor tazobactam significantly reduced the resistance to piperacillin from 73% to 19%. However, the addition of the β-lactamase inhibitor clavulanic acid did not significantly reduce the resistant strains to amoxicillin (from 86% for amoxicillin to 62% for amoxicillin + clavulanic acid) and ticarcillin (from 83% for ticarcillin to 76% for ticarcillin + clavulanic acid). The lowest percentage of resistance was exhibited towards cephalosporin antibiotics (19.76–38.37%) (Figure 2).

However, almost all isolates were resistant to nitrofurantoin (84.88%) and trimethoprim-sulfamethoxazole (51.16%). The resistance rate of isolates against fosfomycin was 27.9% and to colistin 38.4%, and lower percentages of nalidixic acid (37.2%), ofloxacin (36.04%), aztreonam (31.39%) and ciprofloxacin (32.55%) resistant strains were found (Figure 2). 

**Figure 3:**
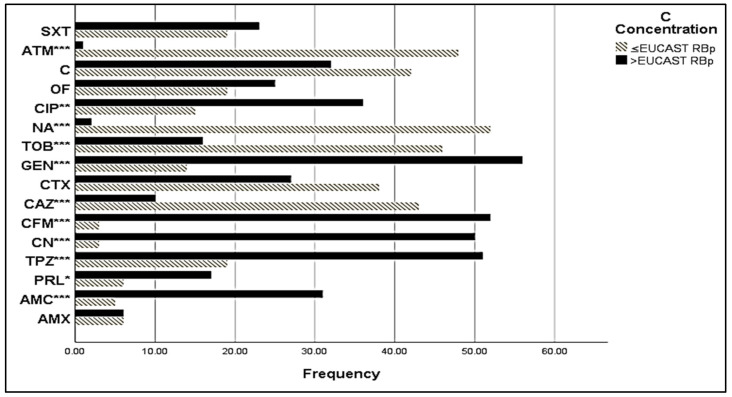
MIC of sensitive *E. coli* isolates. C Concentration stands for Critical Concentration; EUCAST RBp stands for EUCAST Resistant Break point; *: signification (*p* < 0.05); **: signification (*p* < 0.01); ***: signification (*p* < 0.001). AMX: amoxicillin, AMC: amoxicillin + clavulanic acid, TC: ticarcillin, PRL: piperacillin, TPZ: piperacillin + tazobactam, CN: cephalexin, CFM: cefixime, CAZ: ceftazidime, CTX: cefotaxime, GEN: gentamycin, TOB: tobramycin, NA: nalidixic acid, CIP: ciprofloxacin, OF: ofloxacin, C: chloramphenicol, ATM: aztreonam, SXT: trimethoprim/sulfamethoxazole.

Most of the isolates were susceptible to chloramphenicol, gentamycin, amikacin, and tobramycin (86.05%, 81.4%, 83.73%, and 76.75%, respectively) (Figure 2). A high number of *E. coli* isolates had a minimal inhibitory concentration (MIC) value > EUCAST resistance break point (Figure 3), especially for amoxicillin (AMC), piperacillin (PRL), piperacillin + tazobactam (TPZ), cephalexin (CN), cefixime (CFM), gentamycin (GEN), ciprofloxacin (CIP), and trimethoprim-sulfamethoxazole (SXT), with MIC values of >32 mg/L, 8 mg/L, 8 mg/L, 16 mg/L, 1 mg/L, 2 mg/L, and 0.5 mg/L, respectively. The rate of susceptibility was higher towards ceftazidime (CAZ), cefotaxime (CTX), tobramycin (TOB), nalidixic acid (NA), chloramphenicol (C), and aztreonam (ATM) (81.48%, 58.46%, 75.75%, 96.22%, and 58.44%, respectively).

Different antibiotic susceptibility patterns were found among the phylogenetic groups. The most resistant was phylogroup B2 (resistance score: median 14; range 10–30) followed by phylogroup E (resistance score: median 8; range 5–16) and unknown state (resistance score: median 3; range 2–7) (ANOVA test, *p* = 0.001). The most susceptible phylogroups were phylogroup D and Clade I or Clade II. However, no significant correlation was seen between phylogenetic groups and antibiotic resistance; indeed, all phylogenetic groups had a variable resistance to penicillins (26–56.63%). The E groups presented with the highest percentage of resistance to cephalosporins. Moderate resistance of all *E. coli* groups was shown towards trimethoprim/sulfamethoxazole, while the aminoglycosides, especially amikacin, were the most effective antibiotics against all phylogenetic groups.

Further, 69.77% and 23.26% of the examined strains were MDR and XDR strains, respectively. The prevalence of MDR and XDR strains was very significant (*p* < 0.0001) in the adult group. According to phylogenetic groups, the prevalence of MDR strains was higher in phylogroup B1, unknown, and E (100%, 81.81%, and 73.68%, respectively). In contrast, only a few strains (6.98%) were resistant to less than three classes of antibiotics (R) (Table 2). 

### 2.4. Extended β-Lactamase (ESBL) Production and Haemolysin Activity

**Figure 4:**
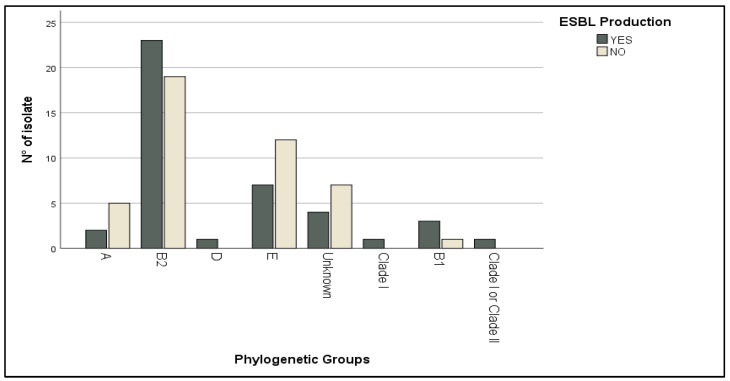
Distribution of ESBL-producing strains according to phylogenetic groups.

The production of ESBL is one of the main mechanisms by which bacteria resist lactam antibiotics. Out of the isolates tested, 48.83% were found to be ESBL-producers while 51.16% were not (Table 2). The ESBL-producing isolates showed greater resistance to β-lactam antibiotics than ESBL-negative, such as amoxicillin (100% vs. 73%, *p* = 0.0002), ticarcillin (98% vs. 68%, *p* = 0.0003), ticarcillin + clavulanic acid (96% vs. 57%), and piperacillin (98% vs. 50%, *p* < 0.0001), and there was a strong, positive, and significant correlation between ESBL production and cefixime (*p* = 0.017) and ceftazidime and cefotaxime (*p* < 0.0001) resistance. Most of the strains did not present hemolytic activity (48.83%), while 22.09% and 29.06% of the isolates featured α-hemolysin and β-hemolysin activity, respectively (Table 2). ESBL producing isolates, as well as strains that produced hemolysin (57.89% for α-hemolysin and 48% for β-hemolysin), were preferentially observed in phylogenetic group B2 more than in other phylogroups (Figure 4).

### 2.5. Correlations between Biofilm Activity, Phylogroup, and Antibiotic Resistance

According to biofilm formation assay results (Table 3), most of the tested clinical strains were weak biofilm producers (71% with *p* value < 0.0001), while other strains did not produce biofilm at all (19%). Moderate (9%) and strong biofilm (1%) producers were in the minority. Biofilm-forming strains were mostly found in the phylogroup B2, although they were mostly weak producers (30%), while the only strong biofilm-forming strain belonged to phylogroup E (*n* = 1, 1.1%).

Weak biofilm production was more commonly found in ESBL-producing strains than in ESBL-negative (42% vs. 29%). There was a statistical correlation between ESBL production and biofilm production (*p* = 0.02) (Table 3).

**Figure 5:**
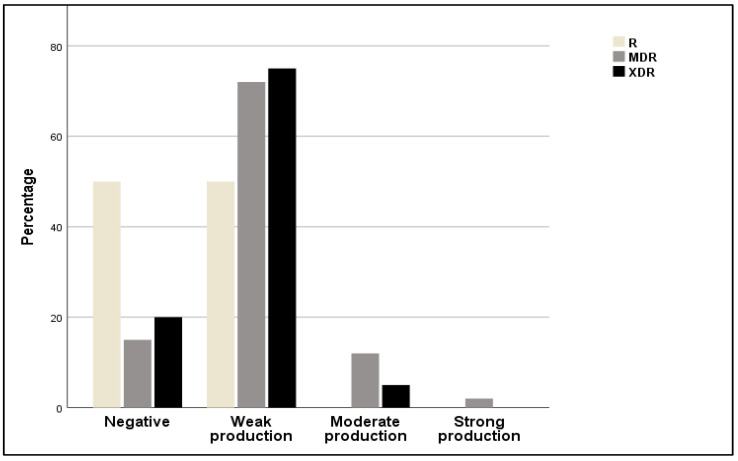
Relative frequencies of resistant (R), multidrug-resistant (MDR), and extensively drug resistant (XDR) strains among non-producers and weak, moderate, and strong biofilm producers.

In the present study, the production of biofilm and antibiotic resistance were analyzed (Figure 5). It was observed that strains possessing resistance to multiple classes of drugs (XDR) exhibited weak biofilm production (75%), or moderate (5%), or were completely unable to produce biofilm (20%). Regarding MDR, most tested isolates had a weak production of biofilm (72%). Furthermore, 50% of resistant strains (R) were not able to form biofilm or produced only weak biofilm (50%). 

**Figure 6:**
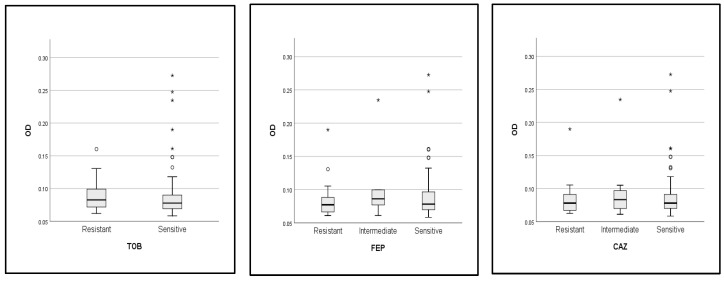

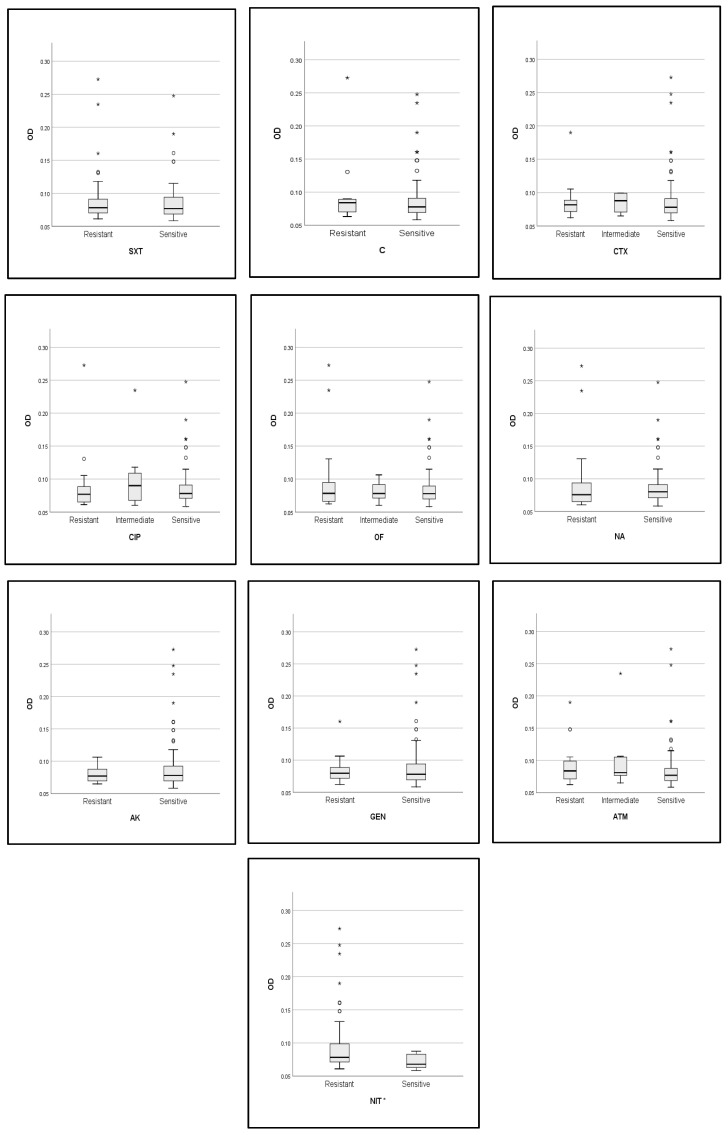
Comparison of OD values of biofilm formation between resistant, intermediate, and susceptible strains. *: *p* value < 0.05, TOB: tobramycin, FEP: cefipime, CAZ: ceftazidime, SXT: trimethoprim-sulfamethoxazole, C: chloramphenicol, CTX: cefotaxime, CIP: ciprofloxacin, OF: ofloxacin, NA: nalidixic acid, AK: amikacin, GEN: gentamycin, ATM: aztreonam, NIT: nitrofurantoin.

In addition, the optical density (OD) of biofilms was compared across the resistance profile (sensitive, intermediate, and resistant strains) for each of the antibiotics tested in this study (Figure 6). The analysis revealed that there was no difference in the OD value of biofilm activity between the resistant, sensitive, and intermediate strains to each of the antibiotics. In this case, the biofilm-forming ability was not associated with the resistance profile of strains; as an exception, the nitrofurantoin-resistant strains produced more biofilms than the sensitive strains with a statistically significant difference (*p* = 0. 012). 

Table 4 displays the percentage distribution of bacteria resistant to each tested antibiotic in weak, moderate, strong, and non-producing biofilm strains. It has been observed that cephalosporin resistance was significantly (*p* = 0.023, with correlation test) decreased for both moderate and strong biofilm-producing strains. On the other hand, weak or non-biofilm producing strains showed a higher percentage of resistance to cephalexin (CN), cefixime (CFM), ceftazidime (CAZ), and cefotaxime (CTX). These findings suggest that the ability to resist this group of antibiotics is inversely proportional to biofilm production. Moreover, with nitrofurantoin, the resistance percentage increased with the biofilm production profile from the non-producing strains to strong producers. In comparison to other resistance categories of antibiotic-resistant strains, there was no significant difference (*p* ˃ 0.05) in biofilm formation.

## 3. Discussion

This study aimed to isolate imipenem-resistant UPECs from patients with UTIs and to characterize them by coupling biochemical and molecular approaches. We were able to confirm the high prevalence of *E. coli* in UTIs [18,19,20], with women being mostly affected, in accordance with previous studies [21,22]. This can be due to anatomical factors like a shorter urethra, making entering the urinary tract easier for bacteria [23,24]. Accordingly to previous research [25,26], UPEC isolates were more prevalent in adults than in the elderly, who can have weaker health conditions due to pathologies like diabetes or kidney stones, medications, and age-related changes in the immune system or the bladder [27]. Various socioeconomic factors, hygiene practices, diets, and lifestyles can also increase the frequency of UTIs, but they were not investigated in the current study.

Most strains causing extraintestinal infections are predominantly categorized into B2 and D groups [12], while commensal isolates are categorized into groups A and B1 [4]. Our findings align with numerous studies that have identified B2 group strains as the dominant type in UTIs. A study on 105 *E. coli* isolates from Slovenian patients with bacteremia of urinary tract origin showed that 51% belonged to group B2, 20% to group D, 15% to group A, and 13% to the B1 group [6]. Another study on 190 urinary *E. coli* isolates in Colombia showed that 46.8% of the isolates belonged to group B2 followed by D group with a percentage of 25.3% [28]. In a study on 228 UPEC in Egypt, 64.6% of the isolates belonged to phylogroup B2, and 18.9%, 10.7%, and 5.7% belonged to phylogenetic groups D, A, and B1, respectively [29]. Similarly in a study for phylogenetic typing urine samples in Korea, the prevalence of uropathogenic *E. coli* belonging to group B2 (77.7%) followed by group D (17.5%), B1 (3.4%), and A (1.4%) [25]. In a study on 113 uropathogenic *E. coli* isolates in Iran, 44.2% of the strains were classified into group B2, 31% into group D, 20.4% into group A, and 4.4% into group B1 [10]. Similarly, studies in India [4,30], Iran [31], and Egypt [32,33], demonstrated that most UPEC isolates from UTIs belonged to the B2 group. Among *E. coli* phylogenetic groups, the B2 phylogroup is believed to be more important than others. This phylogroup is associated with a high evolution of virulence capacity and characteristics, which may cause the spread and persistence of extraintestinal infections representing, therefore, a major public health concern [34,35]. 

Our analysis did not allow the classification of a small percentage of *E. coli* isolates (12.8%). This latter result can be dependent on the recombination of different or rare phylogroups resulting from the combination of the presence and absence of certain genes, as suggested by Boroumand et al. [5]. Phylogenetic group E also had a high prevalence among our strains, as found in a very recent study [36]. However, it should be noted that variations in the source of bacterial isolation, host health state, geographic locations, and genetic variables can affect the distributions and proportions of phylogenetic groupings.

In addition, this study investigated the antibiotic resistance profile of the UPEC isolates, since the spread of antibiotic-resistant strains is a major concern in clinical practice, particularly in developing countries. Due to its high levels of antibiotic resistance, the occurrence of virulence and resistance genes and frequent transmission between humans in different settings and between humans and animals [37,38], our study was focused on imipenem-resistant *E. coli*, since carbapenems are frequently used in hospital setting as first line drugs in the empirical treatment of several bacterial infections in Algeria. In this study, the 86 imipenem-resistant isolates displayed a high percentage of resistance to penicillins, similar to other studies: 78.6% penicillin-resistant urine strains in Uganda and 78.4% penicillin-resistant-UPEC in Mongolia, respectively [23,39]. A high percentage of resistance to nitrofurantoin (84.88%) was also found, while moderate percentages of resistant strains to amoxicillin + clavulanic acid, quinolones, trimethoprim/sulfamethoxazole, and fluroquinolones (32–37.1%) were observed. In Algeria, nitrofurantoin and trimethoprim-sulfamethoxazole are recommended as the first-line therapy, while β-lactams and fluoroquinolones are used as alternative agents in UTI therapy [20,40]. The resistance percentage of the cephalosporin class (19.76–38.37%) was similar to that obtained in Gabon (30–33%) and Rwanda (29.1%) [41,42] and can be linked to the spread and acquisition of the plasmid-borne ESBL genes [43]. The evidence that a lower percentage of isolates was resistant to aminoglycosides (16.2–23.25%), as reported in other studies carried out in Iran (16.7% and 21.8%) [44], could be explained by the limited use of this antibiotic in UTI treatment in developing countries. These results indicate a worrying trend of increased resistance to first-line treatments.

The antibiotic resistance profile of *E. coli* phylogroups showed that B2 groups were more resistant than the other phylogenetic groups. Our finding is consistent with several studies [5,12,45,46,47]. This can be explained by the fact that this phylogroup has a greater ability to exhibit characteristics associated with antibiotic resistance (antibiotic resistance genes), the coexistence of some virulence factors, followed by the acquisition of resistance [6]. On the contrary, many studies have proven that the phylogroup B2 is more sensitive than the other phylogroups (Iran, Taiwan) [48,49]. Social and environmental conditions and the therapy profile of patients may explain this difference. Most of our B2 strains were MDR (69.77%), similar to studies conducted in Egypt and Sri Lanka, featuring 65.17% and 60.3% of MDR strains, respectively [36,50]; this high similarity in percentage of MDR may be due to the similar inappropriate use of antibiotics and poor healthcare infrastructure and management in these developing countries. Several previous investigations have shown that MDR profiles are associated with less virulent strains and non-B2 phylogenetic groups [6]. 

In this study, as already reported [4,51,52], the majority of ESBL-producing and hemolytic strains belonged to B2 phylogroups. The B2 phylogroup‘s increased virulence has been correlated to its ability to persist in the gut microbiota, facilitating the accumulation of virulence and antibiotic resistance genes [9,53].

Biofilms represent a microbial characteristic that protect bacteria against hydrodynamic flow conditions, especially in UTIs and also against host defense mechanisms [54]. In this study, 81.39% of *E. coli* isolates analyzed showed considerable biofilm activity, with 24.8% of the isolates being classified as moderate to strong biofilm producers. This finding is consistent with Gunathilaka et al. (2024) (78% of tested strains were biofilm producers) [36]. Hashemizadeh et al. (2017) found that 74% of the tested strains were biofilm producers in inpatients and 83.4% in outpatients [55]. In another study, Maharjan et al. (2018) found that 21%, 14% and 11% of the strains tested were weak, moderate, and strong biofilm producers, respectively [56]. 

Several previous studies showed that the most of biofilm-forming strains belonged to phylogenetic group B2 [57,58], which is in accordance with our results. Virulence factors, toxin proteins, multi-drug resistance, and ESBL increased in UPEC and is related to phylogroup B2 [59]. The majority of biofilm-producing strains were MDR, confirming the results of a study conducted in Uganda in which 63% of *E. coli* urine isolates were biofilm formers [60]. Similar to our findings, Behzadi et al. (2020) found that there was a significant correlation between ESBL production and biofilm-formation [61].

We found an inverse relationship in 86 *E. coli* isolates between resistance to cephalosporins and biofilm production. Similar results were obtained by Gajdacs et al., who found an inverse relationship between resistance to cephalosporins and biofilm production, and the biofilm producers were less prevalent among third generation cephalosporin-resistant strains [62]. Cepas et al. found that there was an inverse association in biofilm formation ability and resistance to gentamicin and ceftazidime among *E. coli* strains [63]. 

## 4. Materials and Methods

### 4.1. Origin of Isolates and Bacterial Strains

This retrospective study was performed on 86 imipenem-resistant *E. coli* isolates collected from patients with UTIs (all symptomatic infections by uropathogenics involving any part of the urinary tree manifest in many symptoms [64]) 

After 3 years of collection (2021–2022–2023), 402 strains were isolated from different care territories (from the east, west, and north of Sétif province). Among them, 33.8% of the strains were resistant to imipenem, and 86 strains, collected from February to May 2023, were selected for this study from six medical diagnostic laboratories and three hospital laboratories. The eighty-six unique strains of *E. coli* were isolated from urine specimens and collected using standard sterile procedures (only positive cultures with count of 10^5^ Colony Forming Units/mL were taken into consideration for this study). UPEC strains were isolated from both hospitalized and non-hospitalized patients of all age groups with a diagnosis of UTI. After collection, samples were cultured on standard media, including nutrient agar, MacConkey agar, and nutrient broth (TM-Media, Delhi, India) and incubated at 37 °C for 24 h to observe the colony morphology (shape, size, texture, edge and elevation, and opacity). Conventional microbiological methods like Gram staining, and biochemical characteristics, such as IMVIC (Indole test, methyl red test, Voges–Proskauer test, and citrate utilization test), catalase test, urease production, nitrate reduction, motility, triple sugar iron (TSI) test, and gas production were used for *E. coli* identification. Isolated strains were stored in nutrient broth (NB) with sterile glycerol at −20 °C.

### 4.2. Antimicrobial Susceptibility Testing

The Kirby–Bauer disk diffusion method was used for the evaluation of susceptibility in culture media of Muller–Hinton’s agar. Susceptibility testing was performed for 25 antimicrobial drugs (HiMedia Laboratories, Mumbai, India; BioMaxima, Lublin, Poland; BioScan Industrie, Setif, Algeria) including amoxicillin (AMX-25 µg), amoxicillin + clavulanic acid (AMC-20 µg and 10 µg), ticarcillin (TC-75 µg), ticarcillin + clavulanic acid (TCC-85 µg), piperacillin (PRL-30 µg), piperacillin + tazobactam (TPZ-110 µg), cephalexin (CN-30 µg), cefoxitin (CX-30 µg), cefixime (CFM-5 µg), ceftazidime (CAZ-30 µg), cefotaxime (CTX-30 µg), cefepime (FEP-30 µg), imipenem (IMP-10 µg), gentamicin (GEN-10 µg), amikacin (AK-30 µg), tobramycin (TOB-10 µg), nalidixic acid (NA-30 µg), ciprofloxacin (CIP-5 µg), ofloxacin (OF-5 µg), chloramphenicol (C-30 µg), aztreonam (ATM-30 µg), nitrofurantoin (NIT-300 µg), trimethoprim/sulfamethoxazole (SXT-1.25 µg and 23.75 µg), fosfomycin (FF-50 µg), and colistin (CS-10 µg). Antimicrobial susceptibility profiles were determined by interpreting the breakpoints recommended by the European Committee on Antimicrobial Susceptibility Testing (EUCAST) guideline of 2022 [65]; the isolates were defined as susceptible, intermediate, or resistant. In our study R, MDR, and XDR have been used as acronyms (in all manuscripts): R (resistant) is defined as strains that resist less than three classes of antibiotics; MDR (multi-drug resistant) is defined as strains that resist three to six classes of antibiotics; and XDR (extensively-drug resistant) are strains that resist at least seven classes of antibiotics. Nine antibiotic classes were used: penicillins, cephalosporins, carbapenem, aminoglycosides, quinolones, phenicolate, monobactams, polymyxins, and other antibiotics, in particular, nitrofurantoin, trimethoprim/sulfamethoxazole, and fosfomycin. For all susceptible strains MIC determination was obtained through microtitration. The antimicrobial agents and dilution ranges tested for each are presented in (Table 5). The Muller–Hinton broth (TM-Media, Delhi, India), the prepared solutions with a double dilution of antibiotics, and the adjusted inoculum were distributed in wells of microtiter plates and incubated at 37 °C for 24 h. The results were compared with MIC clinical breakpoints published in the EUCAST guideline to determine the resistance profiles. *E. coli* ATCC 25922 was used as the quality control strain.

### 4.3. Detection of Extended Spectrum β-Lactamases Production

UPEC strains resistant to one or more third generation cephalosporins in the Kirby–Bauer disk diffusion test were screened for ESBL production through a confirmatory test. Confirmatory tests were performed using the double-disc synergy test [66]. Briefly, ceftazidime (30 µg), cefotaxime (30 µg), cefepime (30 µg), and aztreonam (30 µg) disks were placed at a 20 mm center-to-center distance of an amoxicillin + clavulanic acid (20 and 10 µg) disk. Samples were considered positive for ESBL when the inhibition zone around any of the cephalosporin discs increased in the direction of the disc containing clavulanic acid, promoting the appearance of either an enhanced or phantom zone. *E. coli* ATCC 25922 was used as the quality control strain.

### 4.4. DNA Extraction and Phylogenetic Grouping by Quadruplex PCR

Bacterial DNA was extracted from 86 *E. coli* isolates using the Direct PCR of Intact Bacteria (Colony PCR) method, described previously [67]. Briefly, after growing the strains in nutrient agar (Oxoid, Milano, Italy) overnight, 1–2 colonies were dissolved in 100 μL of sterile distilled water. The samples were vortexed for 10 s and then incubated at 99 °C for 15 min. Supernatants were collected after centrifugation at 10,000× *g* for 10 min, and pellets were discarded. A 1% agarose gel electrophoresis was conducted to evaluate the quality of the DNA, while the NanoDrop 2000c spectrophotometer (Thermo Fisher Scientific, MA, USA) was used to assess DNA purity and concentration.

Molecular analyses were performed using the primers listed in Table 6. To characterize the 86 *E. coli* clinical strains within the seven phylogroups (A, B1, B2, C, D, E, and F), the method described in Clermont (2013) was used. Primers F1 and R12, described by Coy et al. (2014), were used to amplify the bacterial 16S rDNA gene (approximately 1500 bp fragment). PCR mixes contained 1 Unit of DreamTaq DNA Polymerase (Thermo Fisher), 10 pmol of the forward and reverse primers, 0.2 µM dNTPs in 1× buffer (Thermo Fisher), and 2 µL of DNA sample. The thermal profile used consisted of the initial denaturation step of 5 min at 95 °C, 35 cycles (30 s for denaturation at 95 °C, 30 s of primers annealing at the temperature reported in Table 2 and 30–90 s for extension at 72 °C based on the size of the amplification product), and a final step of extension for 5 min at 72 °C.

### 4.5. Biofilm Production

Biofilm production was assayed in microtiter plates, essentially as described by Stepanovic et al. (2007) [74], with a few adjustments. Briefly, cells were initially grown in brain-heart infusion broth medium BHIB (Liofilchem, Abruzzo, Italy) with glucose. Subsequently, cultures were diluted with fresh BHIB, and turbidity was adjusted to 0.5 McFarland Standard. The bacterial suspensions were incubated for 24 h at 37 °C in 96 well polystyrene microtiter plates. Unattached bacterial cells or planktonic bacteria were then removed from the culture medium by washing the plate with distilled water. Cells adhering to the plate walls were fixed and stained with crystal violet. The absorbance was measured with an ELISA reader (BioTek, El Dorado Hills, CA, USA) at 570 nm (OD570) to estimate the amount of biofilm formed. The experiments were performed in triplicate. The cut-off value (ODc) for judging whether the biofilm had formed was established as the mean absorbance value of the negative control well +3 standard deviation. Strains with a mean OD value ˃ ODc were considered to be biofilm producers. The interpretation of the results was as follows: OD ≤ ODc = not biofilm producer; ODc < OD ≤ 2×ODc = weak biofilm producer; 2×ODc < OD ≤ 4×ODc = moderate biofilm producer; 4×ODc < OD = strong biofilm producer. *E. coli* ATCC 25922 was used as the control organism [74]. 

### 4.6. Hemolysin Production

The production of hemolysin was tested on 5% human blood agar (type A and O) (BioScan Industrie, Setif, Algeria). *E. coli* strains were plated on blood agar plates and incubated at 37 °C for 18–24 h. Following the visualization of the plates, the bacterial strains were classified as α, β, and γ hemolytic: β hemolysis, when the toxin causes the complete lysis of the red blood cells (often referred to as true lysis) producing a clear, transparent area in the blood agar cultures; α hemolysis, when lysis does not occur but the hemoglobin of the red blood cells is reduced to methemoglobin and a brown/green colored area can be observed in blood agar cultures; and γ hemolysis, or non-hemolysis, when no damage to the cells is caused and no change in the agar plate is observed [75,76].

### 4.7. Statistical Analysis

Statistical analysis was performed using the Statistical Package for the Social Sciences software, SPSS (version 26.0). Categories were compared using the chi-square test and Fisher’s exact test. Antibiotic resistance scores were compared between groups using one-way analysis of variance (ANOVA). The Kruskal–Wallis test was used to determine the association between resistance profiles and OD averages. The significance level was set at *p* < 0.05.

## 5. Conclusions

This study demonstrated that imipenem-resistant UPEC strains found in Algerian UTIs belonged mainly to phylogenetic groups B2 and E. Phylogenetic group B2 displayed heightened virulence attributes, including ESBL production, biofilm formation, and hemolysin activity. The majority of the examined isolates demonstrated a weak biofilm-forming capacity. In addition, our analysis showed that β-lactam antibiotics were ineffective against *E. coli* isolates, while aminoglycosides exhibited pronounced efficacy. Furthermore, MDR strains were weak biofilm producers. A variable relationship between antibiotic resistance and biofilm production was evidenced. In conclusion, this study confirms diversity and heterogeneity among imipenem-resistant UPEC strains and the complex association between biofilm production and antibiotic resistance profiles in UTIs caused by UPEC. The detection of the prevalence of phylogenetic groups, antibiotic resistance profiles, and virulence factors among urinary *E. coli* strains in Algeria will help in understanding the epidemiology of urine pathogens in the north-eastern of Algeria and developing the most appropriate treatment and prevention strategies for UTIs, to contain the spread of antimicrobial resistance and to avoid treatment failure in this geographical area.

## Figures and Tables

**Table 1:** Distribution of patients according to hospitalization (inpatients and outpatients), gender and, age (children ≤ 15 y, adults 15–64 y and elderly ≥ 65 y).

	Gender	Inpatient	Outpatient	Total
Adult	M	3 (3.4%)	11 (12.7%)	14 (16.2%)
	F	3 (3.4%)	38 (44.1%)	41 (47.6%)
Adult Total		6 (6.9%)	49 (57.9%)	55 (63.9%)
Children	M	4 (4.6%)	13 (15.1%)	17 (19.7%)
	F	1 (1.1%)	5 (5.8%)	6 (6.9%)
Children Total		5 (6%)	18 (20%)	23 (26.7%)
Elderly	M	0	4 (4.6%)	4 (4.6%)
	F	0	4 (4.6%)	4 (4.6%)
Elderly Total			8 (9.3%)	8 (9.3%)
Total		11 (12.7%)	75 (87.2%)	86 (100%)

**Table 2:** Distribution of phenotypic resistance (R, MDR, and XDR) among UTI strains and association to gender, age (adults, children, elderly), clinical status (inpatients and outpatients), phylogroups (A, B1, B2, Clade I or II, D, E, and unknown), ESBL production, and hemolytic activity.

	R (*n* = 6, 7%)	MDR (*n* = 60, 70%)	XDR (*n* = 20, 23%)	Total (*n* = 86, 100%)
Gender				
M	3 (3%)	26 (30%)	6 (7%)	35 (41%)
F	3 (3%)	34 (40%)	14 (16%)	51 (59%)
Age				
Adult	3 (3%)	37 (43%)	16 (19%)	56 (65%)
Children	2 (2%)	18 (21%)	2 (2%)	22 (26%)
Elderly	1 (1%)	5 (6%)	2 (2%)	8 (9%)
Clinical status				
Inpatient	0	10 (11%)	1 (1%)	11 (13%)
Outpatient	6 (7%)	50 (58%)	19 (21%)	75 (87%)
Phylogroups				
A	1 (1%)	4 (5%)	2 (2%)	7 (8%)
B1	0	4 (5%)	0	4 (5%)
B2	4 (5%)	27 (31%)	11 (13%)	42 (49%)
Clade I	0	0	1	1 (1%)
Clade I or II	0	1 (1%)	0	1 (1%)
D	0	1 (1%)	0	1 (1%)
E	0	14 (16%)	5 (6%)	19 (22%)
Unknown	1 (1%)	9 (10%)	1 (1%)	11 (13%)
ESBL production				
NO	5 (6%)	33 (38%)	6 (7%)	44 (51%)
YES	1 (1%)	27 (31%)	14 (16%)	42 (49%)
Hemolysin activity				
NO	2 (2%)	32 (37%)	8 (9%)	42 (49%)
α-hemolysin	1 (1%)	9 (10%)	9 (10%)	19 (22%)
β-hemolysin	3 (3%)	19 (22%)	3 (3%)	25 (29%)

R: resistant to less than three classes of antibiotics, MDR: multidrug-resistant, XDR: extensively drug resistant; ESBL: extended β-lactamase; M: male, F: female.

**Table 3:** Correlations between the biofilm-forming ability of the *E. coli* isolates, phylogroups, and virulence factors: ESBL and hemolytic activity. (Only *p* values with statistical significance are shown in the table).

	Biofilm Production					
	No (*n* = 16, 19%)	Weak (*n* = 61, 71%)	Moderate (*n* = 8, 9%)	Strong (*n* = 1, 1%)		
					TOT	*p* Value
**Phylogroup**						
A	2.3%	4.6%	1.1%	0.0%	8.1%	
B1	0.0%	4.6%	0.0%	0.0%	4.6%	
B2	11,6%	31.3%	5.8%	0.0%	48.8%	
Clade I	0.0%	1.1%	0.0%	0.0%	1.1%	
Clade I or Clade II	0.0%	1.1%	0.0%	0.0%	1.1%	
D	0.0%	1.1%	0.0%	0.0%	1.1%	
E	3.4%	16.2%	1.1%	1.1%	22.1%	
Unknown	1.1%	10.4%	1.1%	0.0%	12.7%	
**ESBL production**						
ESBL- negative	13.9%	29.1%	6.9%	1.1%	51%	*p* = 0.02
ESBL- producers	4.7%	41.9%	2.4%	0%	49%	
**Hemolytic activity**						
No hemolytic activity	8.2%	35%	5.8%	0%	49%	
α-hemolysin	3.5%	17.5%	1%	0%	22%	
β-hemolysin	7%	18.6%	2.4%	1%	29%	
**Phenotypic resistance**						
R	3.5%	**3.5%**	**0%**	0%	7%	
MDR	10.5%	50%	8.1%	1.2%	70%	
XDR	4.7%	17.4%	1.2%	0%	23%	

**Table 4:** Percentage distribution of antibiotic-resistant strains based on biofilm formation ability.

Antibiotics		Biofilm Production			
		Negative	Weak	Moderate	Strong
Penicillins	AMX	69%	92%	75%	100%
	AMC	50%	61%	50%	100%
	TC	69%	87%	75%	100%
	TCC	69%	79%	63%	100%
	PRL	50%	80%	63%	100%
	TPZ	25%	16%	25%	0%
Cephalosporins	CN	44%	41%	0%	100%
	CX	31%	34%	13%	0%
	CFM	50%	39%	0%	0%
	CAZ	31%	33%	0%	0%
	CTX	13%	25%	0%	0%
	FEP	44%	39%	13%	0%
Carbapenem	IMP	100%	100%	100%	100%
Aminoglycosides	GEN	19%	20%	13%	0%
	AK	13%	20%	0%	0%
	TOB	19%	25%	25%	0%
Quinolones	NA	50%	31%	25%	100%
	CIP	44%	34%	13%	100%
	OF	44%	31%	25%	100%
Phenicolate	C	13%	13%	13%	100%
Monobactams	ATM	25%	34%	25%	0%
Polymyxins	CS	31%	41%	25%	100%
Other	NIT	63%	89%	100%	100%
	SXT	38%	54%	50%	100%
	FF	25%	31%	13%	0%

AMX: amoxicillin, AMC: amoxicillin + clavulanic acid, TC: ticarcillin, TCC; ticarcillin + clavulanic acid, PRL: piperacillin, TPZ: piperacillin + tazobactam, CN: cephalexin, CX: cefoxitin, CFM: cefixime, CAZ: ceftazidime, CTX: cefotaxime, FEP: cefipime, IMP: imipenem, GEN: gentamycin, AK: amikacin, TOB: tobramycin, NA: nalidixic acid, CIP: ciprofloxacin, OF: ofloxacin, C: chloramphenicol, ATM: aztreonam, CS: colistin, NIT: nitrofurantoin, SXT: trimethoprim/sulfamethoxazole, FF: fosfomycin.

**Table 5:** Antibacterial agents and their dilution ranges used in the susceptibility test of *E. coli* isolates.

Antibacterial Agents	MIC Dilution Range (mg/L)	Antibacterial Agents	MIC Dilution Range (mg/L)
Amoxicillin	2–32	Gentamycin	1–16
Amoxicillin + Clavulanic Acid	16–256	Tobramycin	0.25–4
Piperacillin	4–64	Nalidixic Acid	8–128
Piperacillin + Tazobactam	4–64	Ciprofloxacin	0.125–2
Cephalexin	8–128	Ofloxacin	0.125–2
Cefixime	1–16	Chloramphenicol	2–32
Ceftazidime	0.25–4	Aztreonam	0.125–2
Cefotaxime	0.25–4	Trimethoprim-Sulfamethoxazole	2–32

**Table 6:** List of primers with amplicon size, annealing temperature, and the reference.

Target Gene	Primer ID	Amplicon Size (bp)	T °C	Reference
16S rDNA	F1 R12	1500	56	[68]
*chuA*	chuA.1b chuA.2	288	59	[69]
*yjaA*	yjaA.1b yjaA.2b	211	59	[70]
*tspE4.C2*	TspE4C2. 1b TspE4C2.2b	152	59	[70]
*arpA*	AceK.f ArpA1.r	400	59	[69,71]
*arpA*	ArpAgpE.f ArpAgpE.r	301	57	[72]
*trpA*	trpAgpC.1 trpAgpC.2	219	57	[72]
*trpA*	trpBA.f trpBA.r	489	57	[73]

## Data Availability

The original contributions presented in the study are included in the article, further inquiries can be directed to the corresponding authors.
